# Oxidative Stress-Responsive 1 Kinase Catalytic Activity Promotes Triple Negative Breast Cancer Oncogenic Potential

**DOI:** 10.1021/acsptsci.4c00603

**Published:** 2025-02-27

**Authors:** Azeza M. Fdel, Loren Waters, Ira Sharma, Samuel Jones, Julia Gee, John R. Atack, Sourav Banerjee, Youcef Mehellou

**Affiliations:** † Cardiff School of Pharmacy and Pharmaceutical Sciences, Cardiff University, Cardiff CF10 3NB, U.K.; ‡ Medicines Discovery Institute, Cardiff University, Cardiff CF10 3AT, U.K.; § Division of Cancer Research, School of Medicine, University of Dundee, Dundee DD1 9SY, U.K.

**Keywords:** OSR1, inhibitor, breast, cancer, migration

## Abstract

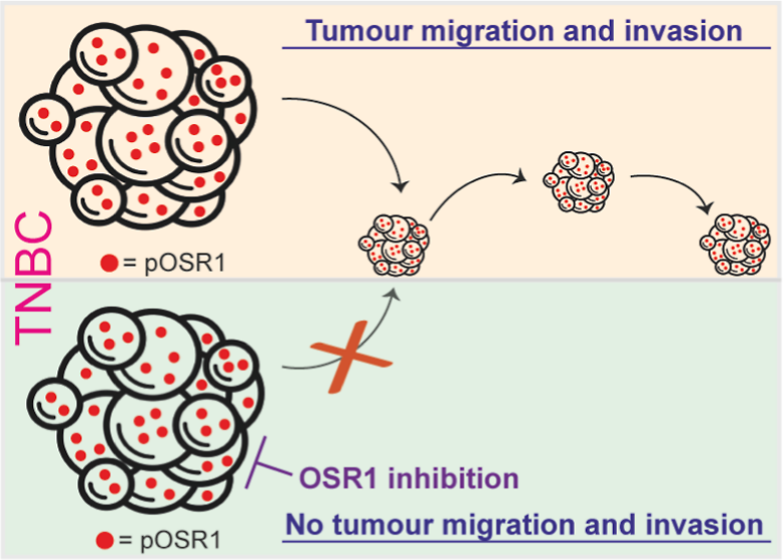

The protein kinase OSR1 has been highlighted as a biomarker for a poor prognosis in breast cancer (BC) patients. To further decipher the mechanism underpinning this, we studied the expression, phosphorylation status, and catalytic activity of OSR1 across a series of BC cell lines. OSR1 was found to be expressed across the various luminal and triple negative BC (TNBC) cell lines studied, although it was only constitutively active in the highly migratory TNBC cell line MDA-MB-231. Although this cell line carries p53 mutations, our data indicated that OSR1 constitutive kinase activity of the OSR1 in MDA-MB-231 was independent of p53. Interestingly, the inhibition of OSR1 had no significant impact on MDA-MB-231 cell viability, but it was found to contribute to its substantial cell migration and invasion, as this was significantly attenuated by the WNK/OSR1 inhibitor WNK463. Analogously, the overexpression of constitutively active OSR1 in the poorly migrating BC cell line MCF7 enhanced its cell mobility. Collectively, our results indicate that the pharmacological inhibition of OSR1 could be a promising novel strategy for preventing the oncogenic potential of TNBC.

The oxidative stress-responsive 1 kinase (OSR1) is a serine/threonine protein kinase that has an established role in controlling ion metastasis.^1−3^ OSR1, and its closely related STE20/SPS1-related proline/alanine-rich kinase (SPAK), with which it shares 68% sequence similarity,^4^ became activated under osmotic stress.^5^ At the molecular level, OSR1 and SPAK become active following their phosphorylation by the upstream with-no-lysine kinases (WNKs 1–4) at the highly conserved threonine residues, T185 for OSR1 and T233 for SPAK, and serine residues S325 for OSR1 and S373 for SPAK (Figure 1).^6^ Notably, the phosphorylation of OSR1 and SPAK kinase domains at T185 and SPAK at T233, respectively, is responsible for turning on their kinase activity.^6^ Active SPAK and OSR1, in complex with the human isoforms^7^ of the scaffolding protein Mouse-Only protein 25 (MO25),^8^ then phosphorylate a series of sodium, potassium, and chloride ion cotransporters such as Na–K–Cl cotransporters 1 and 2 (NKCC1 and 2), NaCl cotransporter (NCC), and KCl cotransporter (KCC) (Figure 1).^1−3^

**Figure 1:**
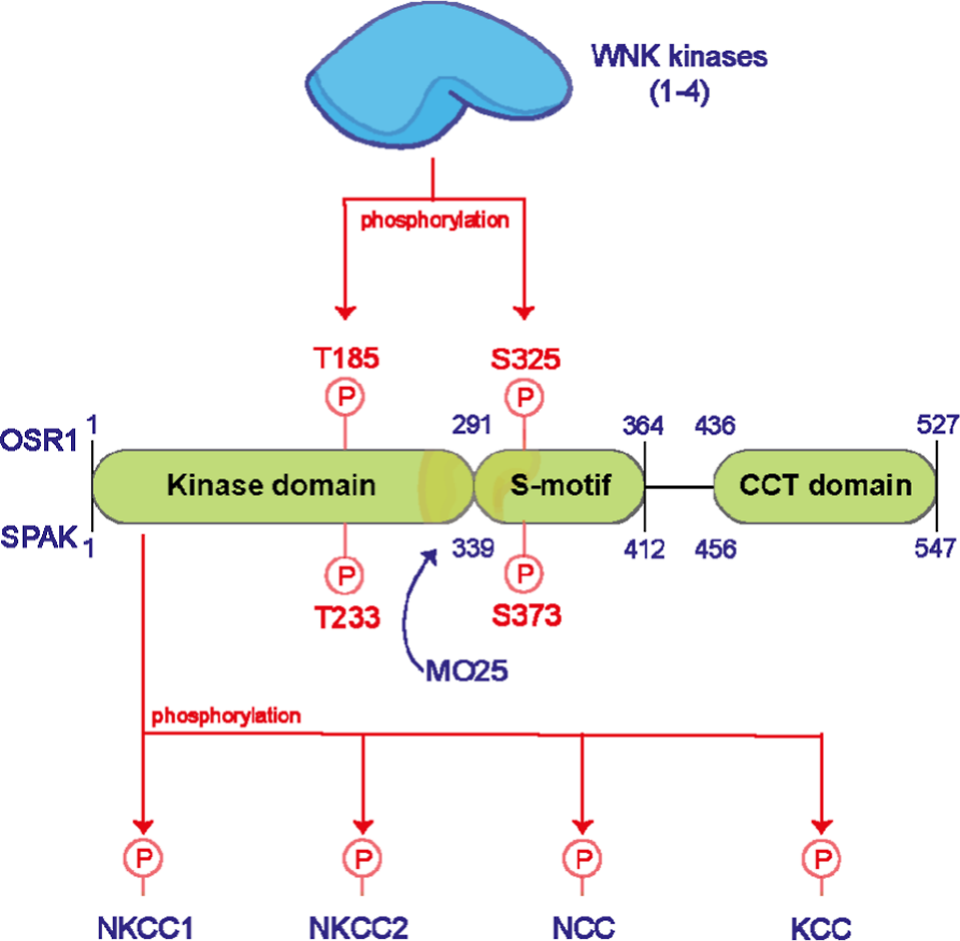
WNK-OSR1 signaling cascade. Human WNKs (1–4) phosphorylate OSR1 and SPAK kinases, with the notable phosphorylation sites being T185 and S325 for OSR1 and T233 and S373 for SPAK. Subsequently, phosphorylated OSR1 and SPAK in complex with the scaffolding protein MO25 phosphorylate a plethora of ion cotransporters such as NKCC1 and 2, NCC, and KCC resulting in the modulation of their function.

Given the involvement of the WNK-SPAK/OSR1 signaling cascade in regulating ion homeostasis, it has been widely studied in the context of hypertension and ischemic stroke.^9−11^ However, recent reports have implicated WNK-OSR1 signaling in the pathogenesis of various cancers,^12,13^ with its involvement in breast cancer (BC), in particular, being validated in patients.^14^ Indeed, it was shown that in BC patients, high expression of OSR1 correlated with estrogen receptor and progesterone receptor negativity, poor prognosis, and lymph node metastasis.^14^ Subsequent work provided a preliminary mechanism that linked OSR1 to the promotion of epithelial-to-mesenchymal transition and metastasis driven by OSR1’s ability to phosphorylate Smad2 and Smad3.^15^ Encouraged by the evidence implicating a role for OSR1 in BC patients, we sought to explore the potential of OSR1 as a target for the treatment of BC. In particular, we focused our attention on Triple Negative Breast Cancer (TNBC), which is characterized by the lack of estrogen or progesterone receptors (ER or PR) as well as the human epidermal growth factor receptor 2.^16,17^ TNBC accounts for 15–20% of all BC cases and is considered to be the most invasive, while its treatment options are very limited in efficacy.^16,17^

## 1 Results and Discussion

**Figure 2:**
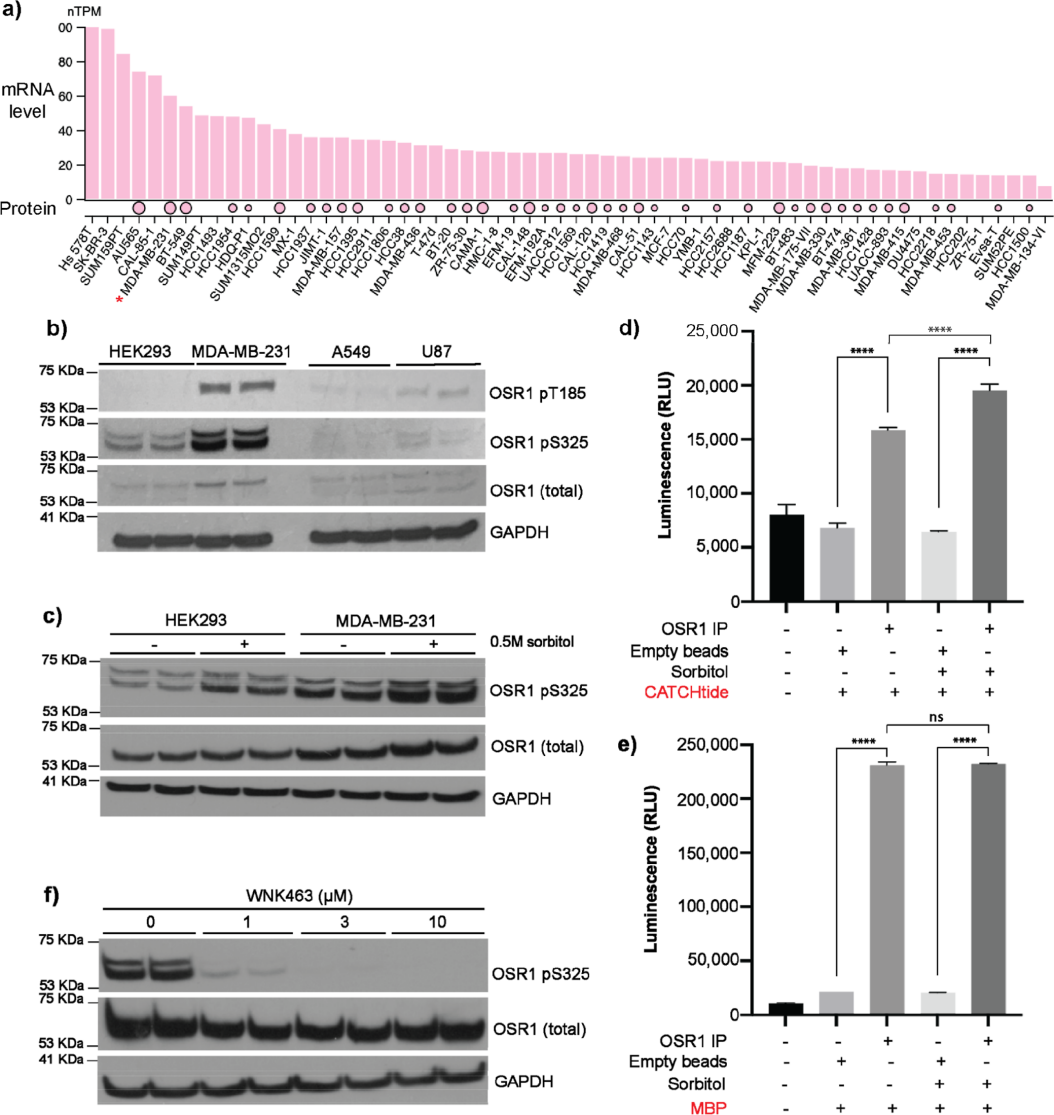
OSR1 is constitutively active in MDA-MB-231 cells. (a) OSR1 mRNA levels (gene name OXSR1) as reported in Human Protein Atlas across the shown BC cell lines measured as transcript per million (TPM). OSR1 protein levels across some of these BC cell lines, as reported in the Human Protein Atlas, are also shown in circles where the size of the circle corresponds to the level of protein expression. (b) Western blot analysis of endogenous OSR1 expression and its phosphorylation at T185 (T-loop) and S325 across multiple cell lines. 20 μg of protein lysates from MDA-MB-231, A549, U87, and HEK293 was probed for total OSR1, OSR1 pT185, OSR1 pS325, and GAPDH as a loading control. (c) Phosphorylation of endogenous OSR1 at S325 in HEK293 and MDA-MB-231 cell extracts either untreated or treated with 0.5 M sorbitol for 20 min. (d,e) Endogenous OSR1 was immunoprecipitated from untreated or sorbitol-treated (0.5 M for 20 min) MDA-MB-231 cells and used in in vitro kinase assays that employ the CATCHtide peptide (d) or myelin basic protein (MBP) (e) as substrates (*n* = 3). ****P < 0.0001, ns: nonsignificant using 2-way ANOVA, mean ± SD from *n* = 3. (f) MDA-MB-231 cells were treated with 1, 3, and 10 μM of the WNK-OSR1 signaling inhibitor WNK463 for 40 min. After cell lysis, 20 μg of total protein extracts was probed using Western blotting to assess total OSR1, OSR1 pS325, and GAPDH as a loading control.

Initially, and as a previous study showed high mRNA expression of the gene of OSR1 (gene name: OXSR1) across three BC cell lines, namely, MDA-MB-231, SKBR3, and MCF-7,^15^ we examined the Human Protein Atlas database for the mRNA and protein expression of OSR1 across 62 BC cell lines. The results showed that the levels of the mRNA of the OSR1 were high in a series of BC cell lines (Figure 2a). Notably, the database also offered some information on the levels of the OSR1 protein across 42 BC cell lines out of the total 62 BC cell lines in the database (Figure 2a). Encouragingly, the data highlighted that both OSR1 mRNA and protein levels need to be high in SKBR3 and MDA-MB231 cell lines, in line with previous experimental findings.^15^ Encouraged by this, we then proceeded to establish the protein expression and activity of OSR1 in the highly aggressive TNBC cell line MDA-MB-231 and two other non-BC cell lines: the lung carcinoma cancer cell line A549 and the glioblastoma cell line U87, as well as the noncancer human embryonic kidney cell line HEK293, which is widely used for studying the WNK/OSR1 signaling pathway.^6,18−20^

Since human OSR1 becomes active following its phosphorylation by the upstream WNKs, mainly at T185 and S325,^6^ we first employed Western blotting to investigate the expression and phosphorylation of OSR1 at T185 and S325 across the cell lines. The results showed that OSR1 was expressed at a very low level across all of the cell lines studied, with its expression in MDA-MB-231 being slightly higher compared to the other cell lines studied (Figure 2b). Strikingly, OSR1 phosphorylation at T185 and S325 was specifically elevated in resting MDA-MB-231 with some marginal phosphorylation in U87 cells (Figure 2b). Given that the phosphorylation of the OSR1 at T185 by WNKs is responsible for turning on its kinase activity, the results shown in Figure 2b suggested that the phosphorylation of the OSR1 was constitutively phosphorylated and potentially active in the TNBC cell line MDA-MB-231. Driven by this observation and driven by our desire to identify new drug targets for the treatment of TNBC, which is currently difficult to treat,^21^ we consequently focused our subsequent studies on MDA-MB-231 cells.

We next investigated whether the observed constitutive OSR1 T185 phosphorylation in MDA-MB-231 was maximal or if it could be induced further upon the stimulation of the WNK/OSR1 signaling cascade. Thus, HEK293 and MDA-MB-231 cells were treated with 0.5 M sorbitol to activate WNK activity leading to their phosphorylation of OSR1 at many sites, most notably T185 and S325.^5^ Our results showed that sorbitol treatment increased OSR1 S325 phosphorylation in HEK293, as expected, and to an extent MDA-MB-231, suggesting that such observed constitutive OSR1 S325 phosphorylation in resting MDA-MB-231 cells was likely not to be maximal (Figure 2c).

Although OSR1 phosphorylation at T185 indicates that the kinase is active,^6^ we sought to further determine OSR1 kinase activity in MDA-MB-231 experimentally. Briefly, endogenous OSR1 was immunoprecipitated from MDA-MB-231 cells, with or without 0.5 M sorbitol treatment. The resulting protein from the various samples was then used in an in vitro kinase assay employing its widely used peptide substrate CATCHtide,^22^ which is derived from its physiological substrate, the ion cotransporter NKCC1, or the generic kinase substrate MBP. Strikingly, endogenous OSR1 immunoprecipitated from untreated MDA-MB-231 exhibited significant phosphorylation of CATCHtide (Figure 2d) and MBP (Figure 2e) in vitro. Interestingly, endogenous OSR1 from MDA-MB-231 cells treated with hypotonic buffer showed kinase activity comparable to that immunoprecipitated from untreated MDA-MB-231 cells (Figure 2d,e) in line with the considerable OSR1 T185 phosphorylation results in this model shown in Figure 2c. The observed constitutive kinase activity of endogenous OSR1 immunoprecipitated from resting TNBC MDA-MB-231 cells is a notable finding because OSR1 generally lacks catalytic kinase activity under resting conditions and only becomes active following osmotic stress or low chloride, hypotonic, conditions.^5^

It is worth noting that the phosphorylation of MBP (10 μM) by OSR1 (Figure 2e) was considerably higher than that observed using CATCHtide (300 μM) as a substrate (Figure 2d). This was also confirmed by a head-to-head comparison of the ability of endogenous OSR1 immunoprecipitated from MDA-MB-231 to phosphorylate 300 μM CATCHtide and 10 μM MBP (Figure S1). Given this OSR1 preferential phosphorylation of MBP by OSR1 compared to that of CATCHtide, it is plausible to suggest that OSR1 may have different substrates in MDA-MB-231 that may be different than those in the established ion cotransporters. Hence, a potential noncanonical WNK/OSR1 signaling in MDA-MB-231 is possible and this deserves further investigation in the future.

In order to provide experimental evidence that the observed constitutive OSR1 phosphorylation in resting MDA-MB-231 cells was WNK mediated, resting MDA-MB-231 cells were then treated with WNK463, an established small molecule pan-WNK inhibitor.^23^ The cells were treated with 1, 3, or 10 μM WNK463 for 1 h. Upon cell lysis and Western blotting, the results showed that in resting MDA-MB-231 cells, the WNK-specific OSR1 phosphorylation site S325 was reduced by the WNK inhibitor WNK463 in a dose-dependent manner (Figure 2f), confirming that the constitutive OSR1 phosphorylation in these cells is WNK mediated. This further supports the earlier finding that the WNK-OSR1 signaling cascade is constitutively active in TNBC MDA-MB-231 cells.

**Figure 3:**
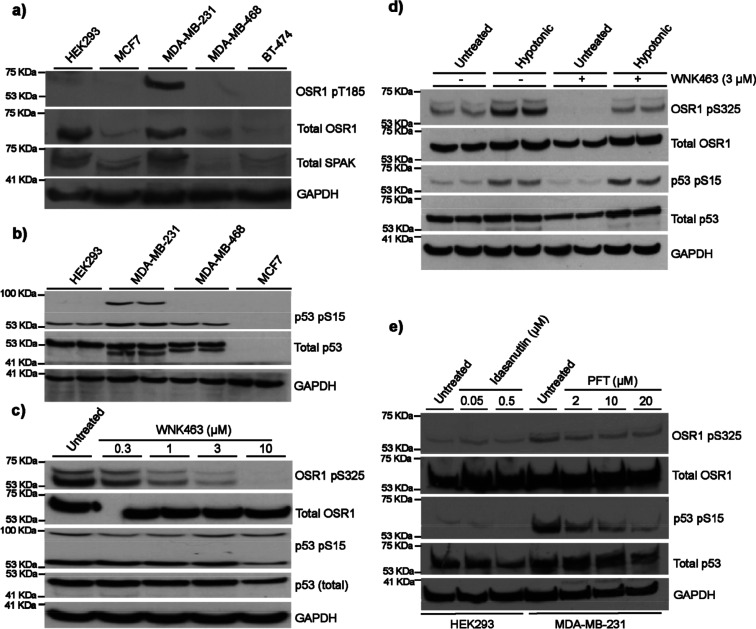
OSR1 kinase activity in BC cells and its relationship to p53 mutations in MDA-MB-231 cells. (a) Western blot was performed to detect the expression and OSR1 T-loop phosphorylation (OSR1 pT185) and expression of its closely related kinase SPAK using 20 μg of protein lysates from four established BC cell lines: MDA-MB-231, MDA-MB-468, MCF7, and BT-474. HEK293 used as a control. (b) Immunoblotting assay examining the phosphorylation of p53 at residue S15 and its expression levels in MDA-MB-231, MDA-MB-468, MCF7 BC cell lines, and the noncancer cell line HEK293. (c) MDA-MB-231 cells were treated with the WNK inhibitor WNK463 at 0.3, 1, 3, and 10 μM for 1 h. After treatment, 20 μg of protein lysates was used in Western blotting for total p53, P53 pS15, total OSR1, OSR1 pS325, and GAPDH as a control. (d) HEK293 cells were treated with basic or hypotonic buffer for 30 min in the presence or absence of 3 μM WNK463 and lysed. Lysates were then subjected to immunoblotting using total P53 and P53 pS15, total OSR1, OSR1 pS325, and GAPDH as a control. (e) HEK293 cells were treated with 0.05 μM or 0.5 μM of the p53 activator Idasanutlin for 8 h, while MDA-MB-231 cells were treated with 2 μM, 10 μM, or 20 μM of the p53 inhibitor PFT for 2 h. Following treatment, cell lysates were subjected to Western blotting to probe for total OSR, OSR1 pS325, total p53, p53 pS15, and GAPDH as a loading control.

Then, we explored whether the OSR1 protein is also constitutively active in other BC cell lines. For this, we used Western blotting to determine OSR1 expression and its T185 phosphorylation as well as the expression of its closely related kinase SPAK across three established BC cell lines, namely, MDA-MB-468 (TNBC; basal A), MCF7, and BT-474 (luminal) compared with MDA-MB-231 (TNBC; basal B).^24^ The results showed that total OSR1 and SPAK levels were more prominent in HEK293 and MDA-MB-231 cells, with almost no visible protein in MDA-MB-468, MCF7, and BT-474 cells (Figure 3a). Strikingly, OSR1 phosphorylation at T185 was only seen in MDA-MB-231 in line with previous results (Figure 2b) but not in MDA-MB-468, MCF7, and BT-474 (Figure 3a), further supporting previously reported data.^15^

Intrigued by this finding and driven by our pursuit of understanding why WNK/OSR1 signaling is constitutively active in MDA-MB-231, but not in the MDA-MB-468, MCF7, and BT-474 cell lines, we noted that MDA-MB-231 carries mutations on the tumor suppressor p53.^25^ This led us to hypothesize that p53 mutations in MDA-MB-231 may have some relationship to the constitutive activation of WNK/OSR1 observed in this model. As such, we performed Western blotting to explore p53 phosphorylation at residue serine 15 (p53 S15) and its expression across MDA-MB-231, MDA-MB-468, MCF7, and the noncancer cell line HEK293. Interestingly, the data showed that p53 is expressed in HEK293, MDA-MB-231, and MDA-MB-468 cells where total p53 appeared as two bands in the latter two cell lines, both of which have been previously reported to carry mutant p53^26^ (Figure 3b). In MCF7 cells, however, it appeared that p53 is not expressed in this cell line (Figure 3b), but high exposure of the Western blot X-ray film showed p53 to be expressed at a very low level in MCF7 (Figure S2) in line with published reports that this model expresses only wild-type p53.^27−29^ In terms of p53 phosphorylation, it was found to be phosphorylated in MDA-MB-231, MDA-MB-468, and HEK293 cell lines at S15 (Figure 3b), which impairs its binding to the negative regulator oncoprotein MDM2 and promotes the accumulation and activation of p53 in response to DNA damage.^30,31^ Notably, p53 S15 phosphorylation was comparatively more elevated in MDA-MB-231 cells, and intriguingly, it appeared as two bands in this model with a higher molecular weight band at ca. 90 kDa (Figure 3b), which could be a dimer version, although this remains unclear. Aiming to understand if there is a relationship between p53 expression and phosphorylation with WNK/OSR1 signaling, we then treated MDA-MB-231 cells with the pan-WNK inhibitor, WNK463, at 0.3, 1, 3, and 10 μM and explored its effect on p53 S15 phosphorylation using Western blotting. The results showed that p53 expression and S15 phosphorylation were not impacted by WNK463 treatment at 1 and 3 μM and were only reduced at the 10 μM dose (Figure 3c) though this is likely to be due to the cytotoxicity of the compound in MDA-MB-231 at 10 μM. For the assay controls, total OSR1 was unchanged following treatment with WNK463 and the phosphorylation of OSR1 S325 was inhibited in a dose-dependent manner (Figure 3c), as expected, and in agreement with data shown in Figure 2f.

Subsequently, we tested whether the activation of WNK/OSR1 signaling has an impact on p53 expression and phosphorylation. Briefly, we treated HEK293 cells with low chloride hypotonic buffer to activate WNK-OSR1 signaling^5^ and investigated its impact on p53 expression and S15 phosphorylation in the presence or absence of 3 μM of the inhibitor WNK463 (Figure 3d). As expected, treatment with hypotonic buffer led to an increase in the level of OSR1 S325 phosphorylation, which was partly suppressed by the treatment with 3 μM inhibitor WNK463 (Figure 3d). Notably, the activation of the WNK/OSR1 signaling did not have any significant impact on p53 expression or S15 phosphorylation (Figure 3d). Together, this suggested that modulation of the WNK/OSR1 signaling, activation or inhibition, does not have an impact on p53 expression and phosphorylation.

Later, we studied whether the activation of p53 by Idasanutlin^32^ or its inhibition by Pifithrin (PFT)-α^33^ would have an impact on OSR1 expression and S325 phosphorylation. HEK293 cells were treated with 0.05 or 0.5 μM of Idasanutlin for 8 h and MDA-MB-231 with 2, 10, or 20 μM of PFT for 2 h. The resulting cell lysates underwent Western blotting for OSR1 S325 phosphorylation (WNK-specific phosphorylation site), OSR1 total protein expression, p53 S15 phosphorylation, and p53 total protein expression with GAPDH as a loading control (Figure 3e). The results showed that as expected, PFT inhibited p53 S15 phosphorylation in a dose-dependent manner, while Idasanutlin exhibited no significant effect on p53 S15 phosphorylation. As for OSR1, these compounds had no obvious effect on the phosphorylation of the OSR1 protein, as judged by the OSR1 S325 protein, or on its total protein expression. This, in addition to data shown in Figure 3c,d, indicated that WNK/OSR1 signaling is independent of p53 S15 phosphorylation and that p53 phosphorylation status and mutation in MDA-MB-231 cells is unlikely to be related to the constitutive activity of OSR1 in MDA-MB-231 cells.

**Figure 4:**
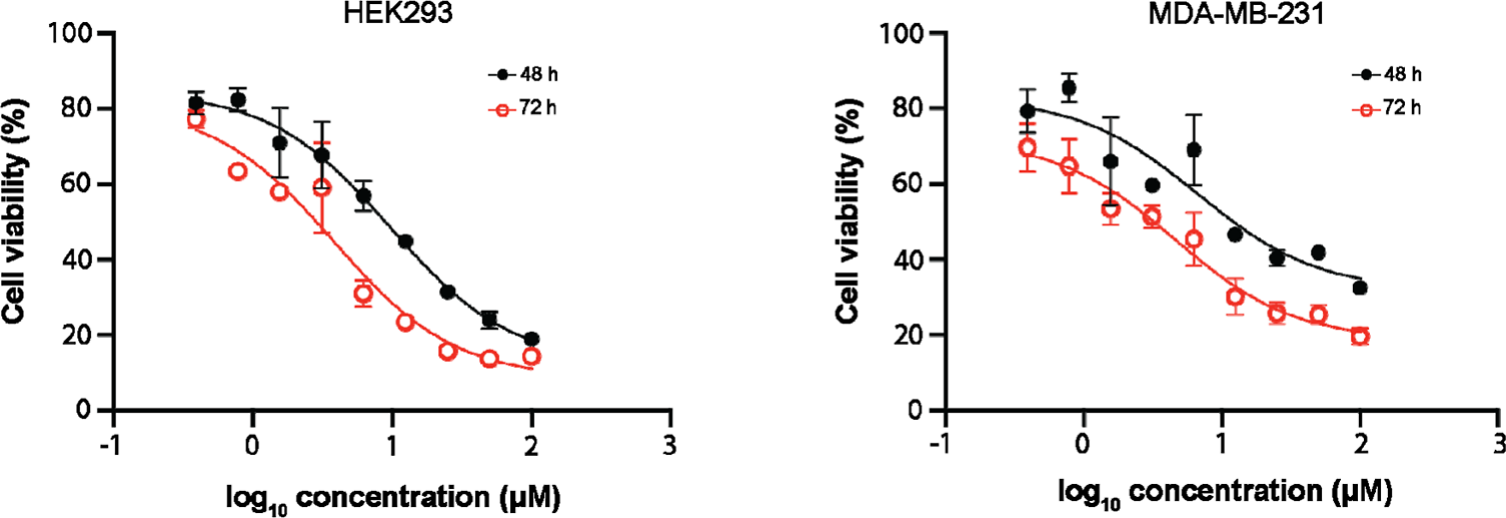
Impact of the WNK-OSR1 signaling inhibitor WNK463 on HEK293 and MDA-MB-231 cell viability. HEK293 and MDA-MB-231 cells were treated with various concentrations (maximum 100 μM) of the WNK-OSR1 signaling inhibitor, WNK463, for either 48 or 72 h. Cell viability was measured using the Promega Cell Viability assay kit. Samples were run in triplicates and the data was consistent across 3 independent repeats.

Following these observations, we then turned our attention to the impact of small molecule inhibitors of the WNK-signaling cascade on TNBC MDA-MB-231 and the noncancer model (HEK293 cells) viability. These cells were treated with a serial dilution of WNK463 (maximum concentration 100 μM) for 48 or 72 h, and cell viability was measured using the commercially available Promega Cell Viability kit. The results showed that WNK463 treatment of MDA-MB-231 or HEK293 cells had a moderate inhibitory effect on their viability (CC_50_: 6.7 μM [48 h treatment] and 4.6 μM [72 h treatment] for MDA-MB-231 and 9.7 μM [48 h treatment] and 3.8 μM [72 h treatment] for HEK293) (Figure 4). The lack of obvious difference of WNK463 impact on the cell viability of MDA-MB-231, which has constitutively active OSR1, and resting HEK293 cells in which OSR1 is not constitutively active was intriguing. One possible explanation could be that in MDA-MB-231, the OSR1 protein levels and high phosphorylation status require higher doses of WNK463 to observe an effect on MDA-MB-231 cell viability as compared to HEK293 cells which have minimal signaling. As WNK/OSR1 inhibition did not impact MDA-MB-231 cell viability akin to what was observed in the noncancer cell line, HEK293 cells, we then investigated the effect of OSR1 inhibition on MDA-MB-231 cell migration using a wound healing scratch assay to begin to determine if the constitutive OSR1 signaling was perhaps more relevant to the aggressiveness of the MDA-MB-231 cells. Indeed, MDA-MB-231 (a basal B TNBC model) is reported to be more invasive and mesenchymal-like compared with luminal cell lines such as MCF7 and BT474 and basal A TNBC models such as MDA-MB-468,^34,35^ all of which we showed had much lower OSR1 activity.

**Figure 5:**
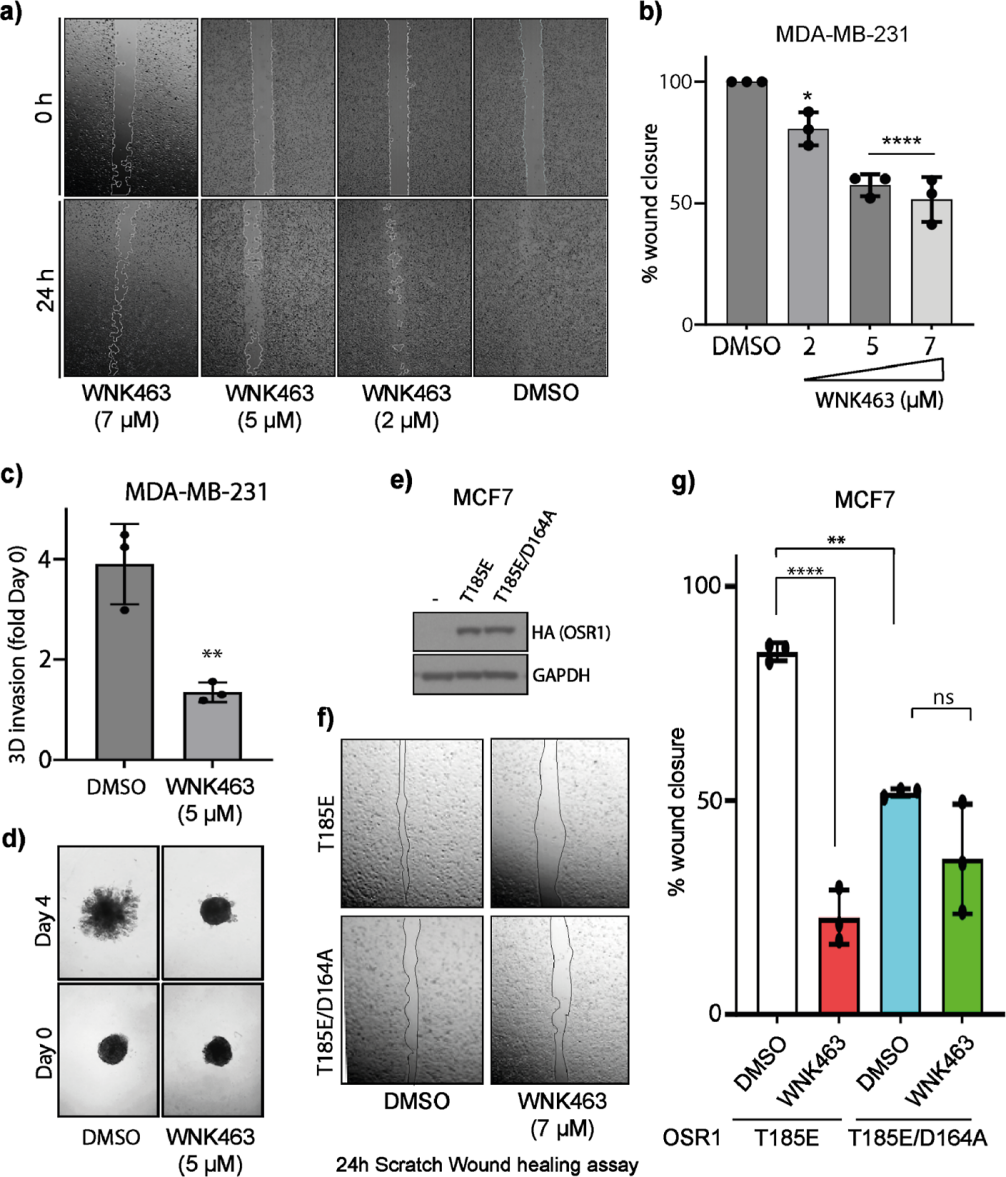
OSR1 kinase activity promotes cell migration and invasion. (a) Representative images of the wound healing assay in MDA-MB-231 cells treated with DMSO (control) or 2, 5, and 7 μM of WNK463 at 0 and 24 h. (b) Bar graph showing percentage wound closure at 24 h in the MDA-MB-231 cell line in control DMSO-treated and WNK463-treated cells. *P < 0.05, ****P < 0.0001 (DMSO- vs WNK463-treated, one-way ANOVA, mean ± SD from *n* = 3 independent experiments). (c) Bar graph showing 3D invasion of MDA-MB-231 cell fold of Day 0 with Control (DMSO treated) and 5 μM WNK463-treated cells. **P < 0.01 (DMSO- vs WNK463-treated, unpaired *t*-test, mean ± SD from *n* = 3 independent experiments). (d) Representative images of 3D spheroid invasion for Day 0 and Day 4 shown below. (e) Immunoblotting analysis was carried out using HA antibody confirming the transfection of constitutively active HA-tagged OSR1 (T185E) or HA-tagged OSR1 kinase inactive (T185E/D164A) overexpression constructs in the MCF7 cell line. (f) Representative photomicrographs of the scratch wound healing assay of the MCF7 cell with OSR1 T185E or OSR1 T185E/D164A overexpression with or without WNK463 (7 μM) treatment after 24 h. (g) Bar graph showing percentage of wound closure in MCF7 cells with overexpression of OSR1 T185E and OSR1 T185E/D164A after WNK463 (7 μM) treatment. ****P < 0.0001, **P < 0.01, ns: nonsignificant (compared to DMSO treated, 2-way ANOVA, mean ± SD from *n* = 3 independent experiments).

To explore this further, we then turned our attention to the potential contribution of OSR1 kinase activity of the OSR1 to MDA-MB-231 migration and invasion. Treatment of MDA-MB-231 cells in a 24 h wound healing scratch assay with titrating concentrations of WNK463 led to a significant inhibition of MDA-MB-231 migration, and this was not observed when the cells were treated with the vehicle only (DMSO), which were highly migratory with virtually complete wound closure (Figure 5a,b). Notably, this finding is similar to previous studies that employed OSR1 shRNAs.^15^ Furthermore, sub-IC_50_ concentration of WNK463 significantly inhibited the invasion of MDA-MB-231 spheroids into the surrounding matrix suggesting an inhibitory effect on both migration and invasion (Figure 5c,d). However, the outstanding question remains whether the contribution of WNK/OSR1 signaling to MDA-MB-231 migration is driven by the activity of the OSR1 kinase activity. To address this question, we performed a wound healing scratch assay using the BC cell line MCF7, which as a luminal BC model is characterized by poor migration, especially compared to the TNBC cell line MDA-MB-231.^36^ MCF7 cells, which do not have constitutively active OSR1 (Figure 3a), were transfected with constitutively active OSR1 (OSR1 T185E) or its kinase dead derivative (OSR1 T185E/D164A) (Figure 5e). We found that while treatment of MCF7 cells expressing constitutively active OSR1 T185E stimulated migration, achieving almost maximal wound healing, introduction of WNK463 significantly inhibited MCF7 cell migration compared with the expression of OSR1 T185E expression alone. The closure seen with the constitutively active OSR1 was significantly less with the kinase dead OSR1, and furthermore, WNK463 had no further effect on the migration of MCF7 cells expressing the kinase dead OSR1 (T185E/D164A) (Figure 5f,g). Collectively, the data provided a novel insight into the notion that the OSR1 kinase activity is a key driver of BC cell migration.

In conclusion, inspired by the emerging link between OSR1 and the pathogenesis of BC, we herein showed that OSR1 is constitutively active in the aggressive TNBC cell line MDA-MB-231 and that this is WNK-phosphorylation dependent. Aiming to understand the reason behind the constitutive phosphorylation of the phenyl groups of OSR1 in MDA-MB-231, we explored whether this is due to the p53 mutations in MDA-MB-231, but our data showed that p53 mutations were independent from the constitutive phosphorylation of the phenyl groups of OSR1 in this TNBC cell line. Encouraged by the OSR1 activity in MDA-MB-231, we then showed that WNK/OSR1 signaling inhibitors did not have a significant impact on MDA-MB-231 viability but significantly decreased migration and 3D invasion ability. This indicated that MDA-MB-231 migration and invasion are driven, at least in part, by the constitutive OSR1 kinase activity. Moreover, we showed that overexpression of constitutively active OSR1 in the BC cell line MCF7 that lacks endogenous constitutive activity of the constitutive OSR1 enhances its migration ability, and this again could be blocked by the WNK/OSR1 inhibitor. Together, the data from this work and others around OSR1’s role of OSR1 in BC indicate that OSR1 inhibitors are unlikely to be effective chemotherapeutic agents for treating BC, but they have a real potential to inhibit BC migration for those tumors with constitutively active OSR1.

Thus, it is envisaged that specific, potent, and safe OSR1 inhibitors could be used in combination with cytotoxic agents and radiotherapy to prevent migration of such BC tumors to secondary sites around the body and potentially inhibit tumor-induced angiogenesis,^37^ which would have favorable impact on BC patient prognosis. Importantly, based on the studies here with MDA-MB-231 cells, this is likely to include a proportion of TNBC cancers where new therapeutic approaches to control their prominent tumor aggressiveness remain much needed.

## 2 Methods

### 2.1OSR1 mRNA Expression

To obtain the mRNA expression data for OSR1 within a panel of BC cell lines, we accessed the Human Protein Atlas (https://www.proteinatlas.org/). First the “cell line” section was selected, and the gene of interest, OXSR1, was entered in the search bar. The results were then filtered to display mRNA expression level specifically for BC cell lines using the “breast cancer” icon. The data was measured in TPM. The results were then further sorted from the highest to the lowest mRNA expression levels, instead of alphabetical order. Relevant data were carefully reviewed including cell line names and expression levels and subsequently exported.

### 2.2 Cell Culture

HEK293, MCF-7, MDA-MB231, MDA-MB468, BT474, U-87, and A549 cells were cultured in DMEM-high glucose, supplemented with 10% fetal bovine serum and 1% pen/Strep (penicillin–streptomycin). Cells were maintained in T-75 flasks at 37 °C and 5% CO_2_. Once the cells reach 80–90% confluency, subculturing into 10 cm cell culture-treated dishes was performed. Following that, hypotonic low chloride buffer treatment was done by replacing the DMEM with the buffer (67.5 mM sodium gluconate, 2.5 mM potassium gluconate, 0.5 mM CaCl_2_/MgCl_2_, 1 mM Na_2_HPO_4_/Na_2_SO_4_, and 7.5 mM HEPES) for 30 min incubation at 37 °C and under 5% CO_2_.

### 2.3 Drug Treatment

Drug treatment was applied on the cells with ∼80% confluency. 10 mM stock concentration was prepared for each compound (in DMSO) used in the experiments. However, the final drug concentration of each experiment varied and is stated in the legend of every experiment.

### 2.4 Cell Lysis

Plates with ∼80% confluency were first washed with PBS and then lysed by adding 300 μL of lysis buffer and scraped. The cells then transferred to Eppendorf tubes to be spun down at 9402*g* for 1 min. The supernatant was transferred to falcon tubes to be stored at −80 °C.

### 2.5 Protein Lysate Preparation

Protein concentration was measured in 96-well plates using the Bradford Assay.^38^ Bovine serum albumin (BSA) was used as the protein standard at concentrations of 0.125, 0.25, 0.5, and 1 mg/mL. In triplicate, 5 μL of each BSA standard concentration was added. Simultaneously, 5 μL of the total protein lysate (also in triplicate) was also added to the plate. Following this, 280 μL of the Bradford reagent was added to each well containing the protein and allowed to incubate for 5 min. Subsequently, absorbance at 595 nm was determined using an Infinite F200 PRO Tecan microplate reader. The absorbance values were analyzed to compute the protein concentration of each sample using linear regression analysis. Initially, the mean absorbance value was calculated from the triplicate samples. A linear regression standard curve was built using Microsoft Excel, plotting absorbance as the *Y* value and protein BSA standard as the *X* value in an *XY*-scatter chart. Once the linear regression curve was established, protein concentration was calculated by determining the *X* value from the linear regression equation *y* = *bx* + *a*, derived from the curve. The resulting *X* value was identified as the sample protein concentration in mg/mL.

### 2.6 Immunoblotting

20 μg of the total protein was loaded per well and subjected to separation on 10% SDS–PAGE gel and transferred onto a nitrocellulose membrane. The membrane then was blocked with 5% skimmed milk in TBST for 20 min at room temperature. The membranes were incubated with the respective primary antibody in 5% BSA or skimmed milk overnight at 4 °C. Following that, the membrane was washed in TBST for 20 min and incubated in the corresponding secondary antibody for 1 h at room temperature. Following a final 20 min TBST wash, the membranes were developed using X-ray film after the ECL reagent application.

### 2.7 Immunoprecipitation

Anti-OSR1 full-length antibodies were linked covalently to Protein G-Sepharose beads using a 1:1 ratio of DMP.^39^ Centrifugation at 847*g*, 4 °C for two min was used to prewash the beads in one volume three times with PBS. Protein G-Sepharose beads were used to preclear MDA-MB-231 protein lysates by incubating them with the beads three times for 10 min at 4 °C. The supernatant was then incubated with 20 μL of the OSR1-Protein G-Sepharose conjugated antibody at 4 °C for 1 h incubation in a rolling shaker. Finally, the immunoprecipitated protein was twice washed in buffer A (10 mM Tris/HCl, pH 8, 0.1 mM EGTA) and lysis buffer to be then used for further assays.

### 2.8 ADP-GLO Kinase Assay

The kinase reaction was carried out in 1.5 mL tubes with a final volume of 25 μL in triplicate. Each reaction had the immunoprecipitated OSR1 by the OSR1-conjugated beads or empty beads as a control. The substrate was 300 μM CATCHtide or 10 μM MBP. Following the preparation of all proteins in kinase buffer (10 mM MgCl_2_, 0.1 mM ATP),^40^ the samples were gently stirred for 40 min at 30 °C while being incubated. The samples were then put into a 96-well plate, where they were developed in accordance with the manufacturer’s instructions (ADP-Glo kit, Promega). The plate was then read using the Infinite F200 PRO Tecan microplate reader to determine the luminesce.

### 2.9 Cell Counting

Cells were counted using a Beckman Coulter counter device. 10 mL of sterile isotonic water was added after 100 μL from 2 mL of cell suspension was transferred to the appropriate cuvette for the counter machine. After inserting the cuvette into the machine’s cuvette holder and pressing the start button, the cell counting process began. The counter determined the cell number for each particle with a size between 9 and 30 μm and displayed it on the monitor. Particle/cell number from the blank was substituted for the displayed number to rectify it (isotonic water only).

### 2.10 Cell Viability

Three replicates of serial dilution of the compound WNK463 were prepared in a flat, transparent 96-well plate (CytOne). WNK463 was made in a stock concentration of 800 μM. Then, 25 μL of the compound stock solution (final concentration: 100 μM) of Trypsin was used to produce a single cell suspension. Following a count of the cells in the cell suspension, 2000 cells (in 75 μL of the cell suspension) were added into each well. In order to account for the media’s absorbance, blank wells (media only) were used. The assay was set up and allowed to run for 48 or 72 h at 37 °C in a humid environment with 5% CO_2_. 15 μL of dye solution was added to each well using a CellTiter 96 Non-Radioactive Cell Proliferation Assay from Promega. The plate was then kept at 37 °C for a further 4 h in a humid environment with 5% CO_2_. 100 μL of the solubilization solution was then added to each well. The absorbance at 570 nm was then recorded on a microplate reader after the plate was left overnight at 37 °C in a humid environment. The percentage of cell viability was then determined using the obtained absorbance and the following formula
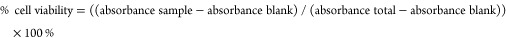


### 2.11 Scratch Wound Healing Assay

Scratch wound healing assay was performed as reported previously.^41^ MDA-MB-231 or MCF7 cells were seeded at a density of 0.6 × 10^6^ cells/ml in ibidi culture inserts (Thistle Scientific). After overnight incubation, the insets were removed, and wells were supplemented with fresh media. Cells were then treated with 0.1% DMSO (control), 2, 5 or 7 μM WNK463. The images were taken on day 0 of treatment and after 24 h. The wound resolution was calculated using the ImageJ MRI_wound_healing plug-in.

### 2.12 Tumour Spheroid Invasion Assay

This assay was performed as described previously.^42^ Briefly, MDA-MB-231 cells were seeded at a density of 1 ×  10^3^ per well in ultralow attachment 96-well round-bottom plates (Nunclon sphere, Thermo Scientific, US) in complete media. After 4 days, media were removed and spheroids were embedded in 100 μL Geltrex (A1413302, Thermo Scientific) per well. After 40 min, each well containing a matrix-embedded spheroid was topped with 100 μL of complete media containing WNK463 (2× concentration for 5 μM final). Images were acquired on day 0 and day 4. Image analysis for invasion was performed using ImageJ 1.X software.

### 2.13 OSR1 Overexpression

HA-tagged OSR1 overexpression in MCF7 cells: MCF7 cells were seeded at a density of 0.3 × 10^6^ cells/mL in 6 well plates. Cells were then transfected with either OSR1 T185E or OSR1 T185E/D164A overexpression constructs using the lipofectamine 3000 reagent (Thermo Fisher Scientific, following manufacturer’s instructions). After 24 h, cells were trypsinized and part of it was used for scratch wound healing and rest for protein extraction. The overexpression was confirmed by Western blot using anti-HA antibody with GAPDH as a loading control.

## Supporting Information Available

Details about the reagents used in this work, buffers, and antibodies; head-to-head comparison of the ability of endogenous immunoprecipitated OSR1 to phosphorylate MBP and CATCHtide; and high-exposure Western blot showing p53 expression across MDA-MB-231, MDA-MB-468, MCF-7, and HEK293 cell lines (PDF)
