# Supplementary material for: Oxidative Stress-Responsive 1 Kinase Catalytic Activity Promotes Triple Negative Breast Cancer Oncogenic Potential
Source: ACS Pharmacol Transl Sci. 2025 Feb 27;8(3):726–35. doi: 10.1021/acsptsci.4c00603 (PMC11915029; doi:10.1021/acsptsci.4c00603)
Supplement: Supplementary file 1 — pt4c00603_si_001.pdf [file pt4c00603_si_001.pdf]

Supporting Information

The Oxidative Stress-Responsive 1 Kinase (OSR1) Catalytic Activity Promotes Triple Negative Breast Cancer Oncogenic Potential

Azeza M. Fdel,[1] Loren Waters,[2] Ira Sharma,[3] Samuel Jones,[1] Julia Gee,[1] John R. Atack,[2] Sourav Banerjee,[3,*] and Youcef Mehellou[1,2*]

[1]*Cardiff School of Pharmacy and Pharmaceutical Sciences, Cardiff University, Cardiff CF10 3NB, U.K.*

[2]*Medicines Discovery Institute, Cardiff University, Cardiff CF10 3AT, U.K.*

[3]*Division of Cancer Research, School of Medicine, University of Dundee, Dundee DD1 9SY, UK*

Email: s.y.banerjee@dundee.ac.uk and MehellouY1@cardiff.ac.uk

Reagents

1. Reagents. CATCHtide peptide (RRHYYYDTHTNTYYLR-TFGHNTRR) was purchased from GLS Peptide synthesis, Shanghai, China. G-Sepharose beads were purchased from Cytiva (CAT No.17061801). Myelin basic protein (MBP) was purchased from Sigma Aldrich (Merck). OSR1 T185E and OSR1 T185E/D164A cDNA clones were purchased from the MRC Protein Phosphorylation Unit, University of Dundee, U.K.

2. Buffers. *Lysis buffer.* The mammalian cells lysis buffer contained 50 mM Tris/HCl (pH 7.5), 150 mM M NaCl, 1 mM EGTA, 1 mM EDTA, 1 mM Na_3VO_4, 50 mM NaF, 5 mM $Na_4P_2O_7$, 0.27 M sucrose, 1% (w/v) Nonidet P40, 1 mM benzamidine, 0.1 mM PMSF, 0.1% 2-mercaptoethanol. *Buffer A* contained 50 mM Tris/HCl (pH 7.5), 0.1 mM EGTA and 1 mM DTT (dithiothreitol). *TBST* (Tris-buffered saline containing Tween 20) was Tris/HCl (pH 7.5), 0.15 M NaCl and 0.2% Tween 20. *Hypotonic low-chloride buffer* contains 67.5 mM sodium gluconate, 2.5 mM potassium gluconate, 0.25 mM $CaCl_2$, 0.25 mM $MgCl_2$, 0.5 mM Na_2HPO_4, 0.5 mM Na_2SO_4 and 7.5 mM HEPES (pH 7.0).

3. Antibodies. OSR1 total antibody (S636B) and anti-OSR1 pS325 (S670B) were purchased from the MRC Protein Phosphorylation and Ubiquitylation Unit at the University of Dundee, UK. Anti-GAPDH rabbit mAb (#2118), anti-rabbit IgG HRP-linked antibody (#7074), p53 total antibody (9282S), and anti-p53 pS-15 rabbit mAb (82530S) were purchased from Cell Signalling Technology. Anti-OSR1 pT185 (A268374) was purchased from antibodies.com. Rabbit Anti-Sheep IgG HRP-linked antibody (ab97130) was purchased from Abcam.

Supporting Figures

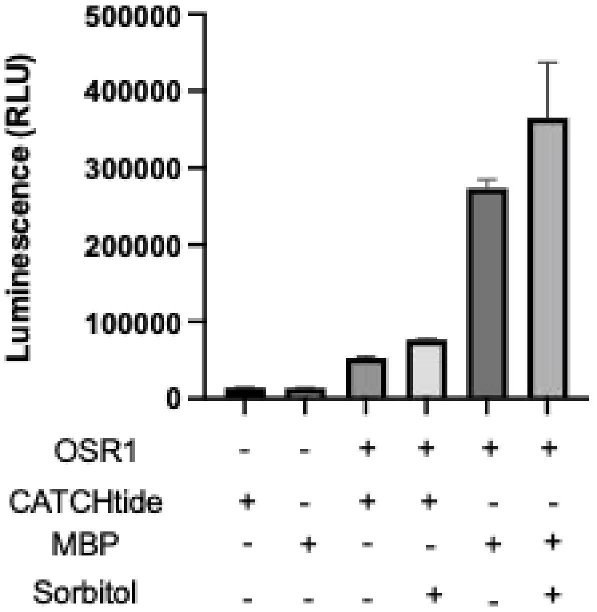

Figure S1. Endogenous OSR1 immunoprecipitated from untreated or 0.5M sorbitol treated (20 minutes) MDA-MB-231 cells using specific OSR1 antibody conjugated to G-protein Sepharose beads. The immunoprecipitated OSR1 was then used in head-to-head in vitro kinase assay that employs CATCHtide peptide (300 μM) or MBP (10 μM) as substrates. The results are shown as the mean activity from a triplicate of samples.

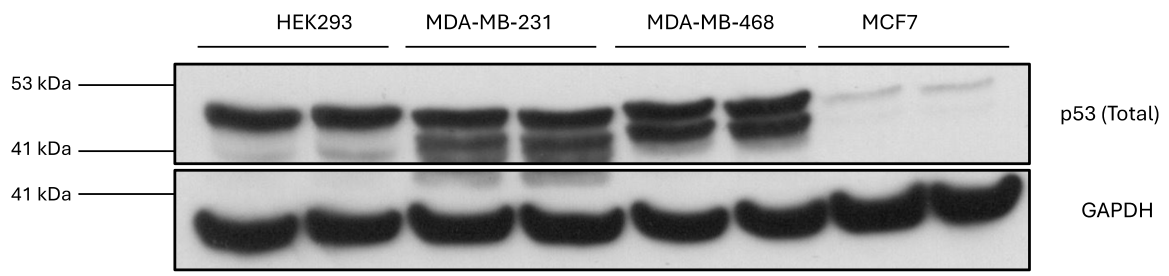

Figure S2. High exposure of the Western blot analysis examining the endogenous expression of p53 across three established breast cancer cell lines: MDA-MB-231, MDA-MB-468, MCF-7, as well as non-cancer cell line HEK293. GAPDH was used as loading control.
